# The Insulin–Urothelial Axis: Evaluating Insulin Resistance as a Convergent Driver of Bladder Cancer Across Diverse Risk Factor Profiles

**DOI:** 10.3390/ijms27093919

**Published:** 2026-04-28

**Authors:** Giovanni Tarantino, Vincenzo Citro, Ciro Imbimbo, Felice Crocetto

**Affiliations:** 1 Department of Clinical Medicine and Surgery, Federico II University Medical School of Naples, 80131 Naples, Italy; 2 Department of General Medicine, Umberto I Hospital, 84014 Nocera Inferiore, Italy; 3 Department of Neurosciences, Reproductive Sciences and Odontostomatology, University of Naples “Federico II”, 80131 Naples, Italy; ciro.imbimbo@unina.it (C.I.);

**Keywords:** bladder cancer, insulin resistance, obesity, type 2 diabetes mellitus, ambient toxicity

## Abstract

Growing evidence suggests that insulin resistance (IR) might be a core, unifying mechanism linking various established risk factors for bladder cancer (BC). While factors like smoking, central obesity, sedentary lifestyle, and high-fat diets are known to increase BC risk, a common thread among them is their role in driving IR due to chronic hyperinsulinemia. Hyperinsulinemia promotes BC development in several ways. It acts as a potent growth factor, stimulating the proliferation and inhibiting the programmed cell death of malignant cells by activating the insulin/IGF signaling pathway. Furthermore, IR is closely associated with chronic low-grade inflammation and oxidative stress, both of which contribute to a pro-tumorigenic microenvironment. This convergence of growth-promoting and inflammatory signals highlights the central role of IR. While more research is needed to fully elucidate these complex interactions, the available data suggest that metabolic interventions aimed at improving insulin sensitivity could be a valuable, modifiable strategy for BC prevention.

## 1. Introduction

In 2022, more than 600,000 people were diagnosed with BC worldwide and more than 220,000 people died from the disease [1]. Considering the predicted 73% and 87% increase in annual BC cases and deaths by 2040, respectively, there is an urgent need to develop and accelerate BC control initiatives for high-risk populations to tackle global BC burden and narrow its geographical disparities [2].

Epidemiological patterns reveal striking demographic disparities: BC is four times more common in men than women and predominantly affects older adults, with over 70% of diagnoses occurring in individuals aged 65 or older [3]. Based on the latest GLOBOCAN data, BC accounts for 3% of global cancer diagnoses and is especially prevalent in the developed world [4]. The metabolic intersection of insulin resistance (IR) and the pathophysiology of BC represent a complex synergy of endocrine disruption, chronic systemic inflammation, and aberrant cellular signaling. IR is defined as a diminished biological response to a given concentration of insulin, primarily affecting glucose uptake in peripheral tissues [5]. Under physiological conditions, insulin binds to the alpha-subunits of the insulin receptor, inducing autophosphorylation of the beta-subunits, which recruit and phosphorylate insulin receptor substrates (IRS-1 and IRS-2) [5,6,7]. The activation of the PI3K pathway is critical for metabolic action. PI3K catalyzes the conversion of PIP-2 to PIP-3, which recruits and activates Akt/PKB. Akt facilitates the translocation of GLUT4-containing vesicles to the plasma membrane, a process mediated by the inhibition of AS160 [6,7]. Chronically elevated glucose levels (glucotoxicity) wear down this system through several mechanisms—(j) Oxidative stress: hyperglycemia increases mitochondrial production of reactive oxygen species (ROS) [8]. (jj) HBP flux: increased flux through the hexosamine biosynthetic pathway leads to O-GlcNAcylation of signaling proteins, impairing their function [6,8]. (jjj) Ectopic lipid accumulation: elevated glucose promotes de novo lipogenesis, leading to the accumulation of diacylglycerols (DAGs) and ceramides. These activate kinases like JNK and IKK\beta, which inhibitory phosphorylate IRS-1 on serine residues, decoupling the receptor from its downstream effectors [5,6]. The tissues primarily involved include skeletal muscle (the site of ~80% of postprandial glucose disposal), adipose tissue (where insulin inhibits lipolysis), and the liver (where insulin suppresses gluconeogenesis) [5,9].

### Pathophysiology and Classification of Bladder Cancer

BC, predominantly urothelial carcinoma (UC), originates from the transitional epithelium [10]. Its pathophysiology is driven by genetic mutations—most notably in FGFR3, PIK3CA, and [11,12]. UC is classified into two distinct clinical pathways: (j) non-muscle invasive BC (NMIBC): Includes stages Ta, T1, and Tis. These are often characterized by FGFR3 mutations and high recurrence rates [10,12]. (jj) muscle invasive BC (MIBC): Includes stages T2 through T4, where the tumor penetrates the detrusor muscle. This phenotype often involves TP53 mutations and carries a high risk of metastasis [11,12]. Diagnosis relies on cystoscopy with biopsy and urinary cytology, while treatment involves TURBT, intravesical BCG, or radical cystectomy for invasive cases [10,11]. Chronic inflammation and the IGF-1R/FGFR3 Axis. BC generates a state of low-grade chronic inflammation through a complex interplay of endocrine, paracrine, and autocrine signals [13]. Tumor cells secrete VEGF to promote angiogenesis and TGF-beta to induce immune evasion [11,13]. Systemic release of tumor-derived cytokines, specifically IL-6, stimulates the liver to produce acute-phase reactants [8,9]. This inflammatory milieu exacerbates IR by activating the JNK and IKK pathways in peripheral tissues [2,5,9]. This creates a vicious cycle where IR leads to compensatory hyperinsulinemia. Since insulin and IGF-1 are potent mitogens, they stimulate the growth and survival of BC cells via the PI3K/Akt and MAPK pathways [9,14]. The IGF-1R/FGFR3 axis functions as a redundant signaling node that bypasses therapeutic inhibition and drives UC progression [5,7]. In BC, FGFR3 mutations trigger constitutive, ligand-independent dimerization [6]. Therapeutic FGFR3 inhibition is frequently circumvented by the compensatory upregulation of IGF-1R [11,12]. (j) Heterodimerization and transactivation: direct physical interaction allows one receptor to phosphorylate the other. Inhibition of FGFR3 often triggers an increase in IGF-1/IGF-2 ligands and IGF-1R expression to maintain PI3K signaling flux [7,11]. (jj) Adapter convergence (IRS-1/2): both receptors utilize IRS-1/2 as primary adapters [5,7]. In systemic IR, elevated insulin/IGF-1 levels saturate these adapters, amplifying oncogenic signals from mutated FGFR3 [6,9]. (jjj) Immune evasion: hyperactive IGF-1R signaling correlates with increased PD-L1 expression [7]. This allows the tumor to suppress T-cell activity, maintaining a “smoldering” inflammation that facilitates tissue remodeling and further mutagenesis [13,14].

The objective of this narrative review was to provide an overview of the literature concerning the mechanistic pathways linking known risk factors to BC pathogenesis and to explore the novel perspective of IR as a potential common pathway. The review was conducted following established methodological standards to ensure transparency and reproducibility. The search strategy utilized keywords and synonyms related to hyperinsulinemia, insulin sensitivity/resistance, BMI, obesity, abdominal adiposity, metabolic syndrome, and BC risk factors. The databases searched were PubMed, Scopus, Web of Science, and Google Scholar. Gray literature sources, such as government reports and conference proceedings, were not considered. This complete electronic search strategy for multiple databases was applied to enable verification and replication of the evidence-retrieval process.

Study selection followed a predefined and transparent workflow, detailing the screening phases, eligibility criteria, and procedures used to resolve disagreements between reviewers. All included studies underwent a structured assessment of risk of bias, providing a clear appraisal of the internal validity of the evidence base. Protocol registration was not undertaken because the scope of the review evolved iteratively during its early conceptualization, making prospective registration impractical and potentially misleading with respect to the final methodological approach. During the preparation of this work the author used AI in order to proofread the manuscript and generate Tables and the Figures. After using this tool, the author reviewed and edited the content as needed and takes full responsibility for the content of the publication.

## 2. Metabolic Dysregulation, Hyperinsulinemia, and Oncogenic Signaling

### 2.1. Excess Adiposity and Bladder Cancer Risk: Current Evidence and Ongoing Controversies

An absolute increase in circulating insulin, or hyperinsulinemia, is associated with obesity [15]. Investigation by de Chuna Agostini et al. evidenced that these factors significantly influence signaling pathways essential for BC development [16], as also evidenced by findings from Huang et al. that revealed that poor glycemic control is associated with poor prognosis in patients with both DM and non-muscle invasive BC [17]. A significant dose–response meta-analysis, which pooled data from 15 cohort studies involving 38,072 BC cases among over 14 million participants, concluded that obesity is associated with a linearly increased risk of BC. This analysis found that for every 5 kg/m^2^ increase in BMI, there was a 4.2% increased risk of BC. When comparing BMI categories to normal weight, the pooled relative risks were 1.07 for pre-obese individuals and 1.10 for obese individuals [18].

A study comprehending 17,777 men newly diagnosed with BC analyzed data from the National Health Insurance System and National Health Checkups databases in South Korea with the aim of assessing the association between metabolic health status and the incidence of BC. When compared to the metabolically healthy-normal-weight group, the multivariable-adjusted hazard ratios (HRs) were higher in other categories: metabolically obese-obese individuals had the highest HR at 1.3; the metabolically obese-normal-weight group showed an HR of 1.18; even the metabolically healthy-obese group exhibited a slightly elevated HR of 1.07. These findings indicate that both obesity and metabolic dysfunction, i.e., the presence of at least three metabolic syndrome components, contribute to an increased risk of BC in men [19]. A previous meta-analysis of 14 prospective cohort studies, encompassing 12,642 BC cases, investigated the relationship between BMI and BC risk. The dose–response analysis revealed a nonlinear positive association between BMI and BC risk showing a summary relative risk (RR) of 1.03. This suggests that for every 5 kg/m^2^ increase in BMI, there is an approximate 3% rise in BC risk, with this increase being particularly notable when BMI exceeds 30 kg/m^2^ [20]. Beyond overall BMI, emerging research also points to specific fat distributions, such as abdominal subcutaneous adipose tissue and the ratio of abdominal subcutaneous to gluteofemoral adipose tissue, as potentially increasing BC risk [21].

A more recent meta-analysis of eleven cohort studies demonstrated a statistically significant association between obesity and an increased risk of BC in the overall study population with a RR of 1.10. Furthermore, among the nine studies that controlled for the confounding effect of cigarette smoking, the pooled RR for BC in obese individuals remained statistically significant at 1.09 [22]. Interestingly, patients with non-muscle invasive BC, who had general or abdominal obesity, did not experience a higher risk of recurrence, but these forms of obesity might be connected to an increased risk of the disease worsening [23,24]. Among the metabolic syndrome components, central obesity was positively associated with BC (HR = 1.39) [25]. Nevertheless, BMI warrants further investigation when making therapeutic decision [26]. Unexpectedly, research indicates that a higher BMI could be associated with better survival rates for some individuals with BC, suggesting that obese patients may have a more favorable prognosis, according to the obesity paradox [27].

Until recently major clinical guidelines, including those from the American Urological Association and the Society of Urologic Oncology, did not consider obesity to be an established risk factor for BC [28]. For instance, the International Agency for Research on Cancer (IARC) Working Group considered the evidence “presently inadequate” for BC, which is a stance that contrasts with other experts who have more recently suggested an increased risk [29]. Similarly, the World Cancer Research Fund/American Institute for Cancer Research report on BC, which synthesized findings from numerous prospective cohort studies, also judged the evidence for an association of obesity with BC risk as limited and inconclusive [30]. In line with the American Cancer Society, selecting what types of cancer are linked with excess body weight, BC is not included [31]. In parallel, according to the 2020 report from the IARC, there is strong evidence that obesity increases the risk of 13 different cancers; however, this association was not found for BC [32]. This position of scientific societies conflicted with previous studies.

### 2.2. Established Yet Incompletely Defined Risk Factors in Bladder Carcinogenesis

Beyond the primary influence of high-potency carcinogens, the development of bladder malignancies is increasingly linked to established yet nebulous factors such as chronic inflammatory signaling, metabolic disturbances, and micronutrient imbalances, though the specific molecular underpinnings and regulatory disruptions that facilitate this neoplastic progression have yet to be fully mapped. After reviewing 1496 articles, researchers identified several key factors influencing BC risk. They found significant increases in risk associated with: smoking (cigarettes, pipes, or cigars), obesity, high consumption of processed meat. Conversely, the study found a lower risk linked to increased physical activity, higher body levels of selenium, vitamin D, vitamin A, vitamin E, and folate. Certain occupations also carried a higher risk, with tobacco workers, dye workers, and chimney sweeps showing the highest associations. The likelihood of individual factors causing BC varied widely, from 4% to 68% [33]. A summary of modifiable and non-modifiable risk factors, and their molecular mechanisms are provided in Table 1. The biochemical pathways with pathological results are shown in Table 2.

### 2.3. The Metabolic Catalyst: Evaluating Tobacco’s Role in Inducing Insulin Resistance and Accelerating Bladder Cancer

Beyond its direct mutagenic effects, tobacco smoke functions as a potent metabolic disruptor that triggers systemic IR, establishing a pro-tumorigenic milieu where hyperinsulinemia and altered glucose homeostasis act as accelerated drivers of bladder carcinogenesis, by the overproduction of bioavailable growth factors and the subsequent constitutive activation of intracellular mitogenic cascades.

Smoking is a major cause of BC [57]. Recent findings from the NIH-AARP Diet and Health Study (started in 1995) show that current smokers face a higher risk of BC compared to never-smokers. This increased risk is more pronounced than what was observed in studies from earlier cohorts (1963–1987), suggesting that the link between smoking and BC risk in the U.S. has strengthened over time [58,59,60]. Studies indicate that smokers with a higher BMI often consume more cigarettes daily and may experience a greater level of nicotine dependence than smokers who are leaner [61]. Among 499,504 adults studied, a clear link was found between smoking and obesity. The more a person smoked, the higher their risk of obesity. Additionally, the risk of obesity for former smokers decreased the longer they had been smoke-free. Notably, former heavy smokers were 60% more likely to be obese than former light smokers [62].

Data from the Korea National Health and Nutrition Examination Survey were analyzed to explore the relationship between smoking and IR, which was defined by the triglyceride-glucose index (TyG). Participants were classified based on urinary metabolite levels into continuous-smokers, past-smokers, current-smokers, and non-smokers. Multiple logistic regression indicated that continuous-smokers and past-smokers (both men and women) had an increased risk of high IR. This observation suggests that smoking cessation could be protective against insulin resistance [63].

Nicotine appears to cause IR in skeletal muscle by activating the mTOR pathway. This suggests that developing treatments to block mTOR activation in skeletal muscle could help prevent IR in people who cannot quit smoking or are regularly exposed to secondhand smoke [64]. Research indicates that both active smoking and exposure to secondhand smoke are associated with impaired glucose tolerance and impaired fasting glucose [65]. Indeed, high prevalence of e-cigarettes was noted among the obese population [66], but the malignant potential of e-cigarettes for BC remains unknown and is likely less than that of combustible cigarettes [67].

### 2.4. Focus on Endocrine Disruptors: Microplastics

Very recent investigation identified a statistically significant spatial clustering of BC incidence. This clustering was predominantly observed in geographical areas exhibiting elevated chemical risk-screening environmental indicator scores, particularly those correlated with documented bladder carcinogens and the presence of microplastic (MP) waste [68]. The demonstrated tumorigenic potential of bisphenol (BP) A and BP S, absorbed onto MP surfaces, coupled with their established ligand-receptor binding affinities and the presence of cognate receptors within the bladder urothelium, collectively suggest a plausible mechanistic involvement of these BPs in the pathogenesis of BC [69].

The precise implications of MP exposure on human health remain an area of ongoing investigation. However, laboratory animal models and cell culture studies collectively indicate that these xenobiotics may contribute to the etiology of obesity through multifaceted mechanisms [70].

Polystyrene (PS) microplastics and nanoplastics have been shown in recent laboratory studies to cause IR in skeletal muscle cells. This happens because the MP lead to mitochondrial dysfunction, which in turn generates an excess of mitochondrial ROS [71].

For better understanding of the health hazards posed by MP, past studies in mice show that PS can worsen metabolic disorders like IR. The effect, observed when comparing mice on a normal diet to those on a high-fat diet, was obtained by disrupting gut microbiota, leading to pro-inflammatory responses [72]. Beyond the physical properties of the MP themselves, plastic additives constitute a significant concern as co-contaminants. Numerous such additives, including organotins, phthalates, and various toxic metals beyond bisphenols, are recognized for their capacity to modulate adipocyte differentiation and interfere with the expression and function of proteins crucial for lipid and glucose metabolism [73]. These additives activate specific cellular components, including nuclear receptors, peroxisome proliferator-activated receptors (alpha, beta, and gamma), and the retinoid X receptor. This activation, in turn, triggers a range of negative effects such as oxidative stress, cell toxicity, harm to the immune system, disruption of thyroid hormones, and changes in fat cell development and energy production [74]. Notably, BP A, a ubiquitous monomer in plastic synthesis, is a well-established endocrine-disrupting chemical. Exposure is positively associated with IR in humans [75]. Such endocrine disruption can profoundly impact metabolic regulation and predispose individuals to weight gain. The escalating global prevalence of overweight and obesity represents a substantial public health challenge, given their strong association with an increased risk of severe co-morbidities, including but not limited to type 2 diabetes mellitus (T2DM) and various neoplastic conditions. The promotion of SOCS-3 expression by BP A may inhibit insulin signal transduction, thereby contributing to the development of IR [76].

### 2.5. Environmental Catalysts: How Air Pollution Fuels the Insulin Resistance–Bladder Cancer Connection

*The “Double Hit” Titles* Researchers conducted an umbrella review of seven studies (five meta-analyses and two systematic reviews) to determine how air pollutants affect obesity. They examined the impacts of common pollutants like PM1, PM2.5, PM10, NO2, SO2, and O3. Most studies indicated that exposure to air pollution was linked to a higher risk of obesity, although the effects varied among different pollutants [77]. A systematic review of 37 studies and meta-analysis of 21 epidemiological studies suggests a significant connection between exposure to air pollution and an increased risk of urological cancers. Specifically, authors found that for every 5 µg/m^3^ increase in PM2.5, the risk of BC rises by 7%. Similarly, a 10 µg/m^3^ increase in NO2 is associated with a 4% higher risk of BC. These findings highlight the potential for public health interventions. If these links are indeed causal, lowering PM2.5 levels to 5.8 µg/m^3^ could lead to a reduction of 1.5 to 27 urological cancer cases per 100,000 people (age-standardized rate) in the countries most affected by high PM2.5 and urological cancer burden [78].

It is not fully understood how environmental air pollutants cause cancer, but the dose and duration of exposure play a role. Carcinogens found in air pollution, whether individually or in mixtures, disrupt several molecular processes. They can cause direct damage or indirect damage through systemic inflammation and oxidative stress. This leads to the inactivation of tumor suppressor genes, the activation of oncogenes, and changes in the cell cycle dependent on p53 activation. Additionally, these pollutants can cause energy dysregulation, chromosome instability, inhibition of apoptosis, and increased cell proliferation in somatic cells. This information was reviewed in recent article [79]. Evidence suggests ambient PM2.5—even at low concentrations—can diminish metabolic insulin sensitivity. This lends plausibility to the idea that air pollution exposure potentiates T2DM development [80]. To corroborate earlier findings, long-term exposures to PM2.5, PM10, NO2 or SO2 are indeed associated with the odds of IR. Among the analyzed pollutants, inhalable particulate matters appear to exert greater impacts on IR [81].

### 2.6. From Tap to Tumor: Is Contaminant-Induced Insulin Resistance the Missing Link in Water-Borne Bladder Carcinogenesis

Groundwater contamination by arsenic has emerged as a significant worldwide issue, posing a severe health risk to millions [82]. Arsenic exposure increases blood sugar levels and the risk of hyperglycemia by increasing IR. This effect is more pronounced in females than in males [83].

A comprehensive review of 30 years of epidemiological research, encompassing 40 qualifying studies, reveals that the majority of these (28 out of 40) found a clear link between arsenic in drinking water and BC. Specifically, meta-analyses predicted the risk of BC incidence to be 2.7, 4.2 and 5.8 times higher at arsenic levels of 10, 50, and 150 µg/L. This means that at an exposure level of just 50 µg/L, there was an 83% probability of increased BC incidence and a 74% probability of higher mortality from the disease [84].

### 2.7. Evaluating Disinfection Byproducts as Metabolic Disruptors in the Bladder Cancer Pathway

A statistically significant difference was observed in female serum cholesterol levels, with higher concentrations found in communities with chlorinated water supplies [85]. On the contrary, short-term exposure to chlorinated drinking water at 20 ppm appears to have no significant impact on parameters of lipid in healthy humans [86]. Most interestingly, daily intake of water with fluoride concentrations >1.5 mg/L produces IR [87]. One meta-analysis involving over 17,000 individuals from European and North American populations suggests that drinking chlorinated water may increase the risk of BC. In this analysis of six case–control and two cohort studies, men who ever consumed chlorinated water had a 40% higher chance of developing BC while women drinking water showed a 20% higher chance. The risk appeared to be dose-dependent, with a 40% increased risk for long-term exposure. Furthermore, the study indicated a linear increase in risk over time, with a 27% increase after 40 years of exposure for both sexes combined [88]. The interaction of disinfectants with wastewater’s organic constituents can yield byproducts, including trihalomethanes (THM). Of the sixteen BC susceptibility genetic variants identified via genome-wide association studies, a single nucleotide polymorphism at locus rs907611, situated within the lymphocyte-specific protein 1 region at 11p15.5, exhibited the most robust association with BC risk, especially with elevated THM exposure via potable water [89].

In multivariate models adjusted for potential confounders, participants with a BMI ≥ 30 had significantly higher odds of T2DM (OR = 8.42) compared to those with normal weight, via insulin signaling disruption. Similarly, individuals in the highest tertile of urinary brominated (Br)-THM levels had nearly four times the risk of T2DM (OR = 3.99) compared to those in the lowest tertile. Furthermore, among participants with a BMI ≥ 25, urinary Br-THM concentrations were significantly elevated in diabetic individuals compared to healthy controls [90]. Consuming contaminant-free water and increasing intake of plant-based foods can help prevent BC [91].

### 2.8. Does Sleep Affect Bladder Cancer Risk?

Insufficient sleep significantly perturbs the intricate network of hormones controlling appetite. Sleep restriction is associated with a decrease in circulating levels of leptin, an adipokine with satiety-promoting effects, and a concurrent increase in ghrelin concentrations, a potent orexigenic peptide. This hormonal imbalance, coupled with extended sleeplessness providing increased opportunities for caloric intake, is hypothesized to promote hyperphagia and subsequent obesity [92]. Sleep deprivation, whether total or partial, initiates a cascade of physiological alterations that contribute to metabolic dysfunction. This includes an elevation in sympathetic nervous system activity and circadian disruption of endocrine rhythms, specifically observed as increased nocturnal cortisol levels and elevated diurnal GH concentrations. These neuroendocrine shifts collectively contribute to compromised metabolic homeostasis, primarily through the induction of IR and diminished glucose tolerance [93]. Beyond direct metabolic and appetite-related effects, chronic sleep reduction and its sequelae, such as somnolence and fatigue, are implicated in reducing overall energy expenditure. This reduction is attributed to decreased participation in gut dysbiosis [94], voluntary physical activity and a reduction in non-exercise activity thermogenesis [95]. The aforementioned deleterious effects are frequently exacerbated in individuals who are overweight or obese, particularly in the presence of sleep-disordered breathing (SDB). SDB, including obstructive sleep apnea (OSA) central sleep apnea, as well as sleep-related hypoventilation and hypoxemia, is a recognized independent risk factor for IR, thus accelerating the progression of metabolic derangements initiated by sleep loss [96,97]. A study including 380,042 UK Biobank participants showed that both healthy sleep and lifestyle patterns were significantly associated with a reduced risk of BC (HR = 0.61), compared to those with unhealthy patterns [98]. Epidemiological evidence from large community studies indicates that obesity is a strong determinant but not a prerequisite for OSA; the greater proportion of people with OSA have a BMI in the non-obese range [99]. Data from 12 studies since 1814, involving over 9.2 million participants, show a significant link between OSA and an increased risk of BC. A quantitative analysis of nine of these studies found that OSA patients had a 76% higher risk [100].

### 2.9. Meat-Centric Diet, Metabolism, and Malignancy: A Hypothetical Framework for Nutrient-Induced Insulin Resistance in Bladder Cancer

It is biologically plausible that what individuals eat can affect BC risk. This is because both helpful and harmful substances from diet pass through the urinary tract and come into direct contact with the bladder epitelium. Despite this, studies examining the link between diet and BC have often shown conflicting results [101].

To understand the relationship between meat consumption and BC, researchers analyzed five cohort studies involving over one million participants and eight case–control studies with more than 27,000 participants. The findings suggested different impacts for red meat and processed meat. Case–control studies indicated that for every 100 g daily increase in red meat consumption, the risk of BC rose by 51%. However, cohort studies did not observe this association with red meat. When looking at processed meat, a combined analysis of both study types showed a 20% increase in BC risk for every 50 g daily increase. The hypothetic mechanism consists of the generation of low ppb levels of mutagenic/carcinogenic heterocyclic amines, during frying and grilling [102].

Furthermore, a systematic review of 21 studies and a meta-analysis of 18 studies (encompassing a massive 1,135,661 participants) points to a strong connection between red and processed meat intake and obesity. The meta-analysis, which included 113,477 individuals, concluded that higher consumption of these meats is a risk factor for obesity, increasing the odds by 37% [103].

Evidence from various populations indicates that the consumption of meat, especially processed and unprocessed red meat, is a significant risk factor for T2DM [104]. These findings suggest that a reduction in meat consumption is a crucial public health measure that should be incorporated into dietary guidelines. High to moderate consumption of meat is linked to a higher risk of IR in women who do not have T2DM. Reducing the amount and changing the type of meat people eat could help lower the chances of developing IR [105]. Meat consumption affects blood sugar and insulin levels through several key mechanisms: nitrosamines can harm the beta cells in the pancreas; saturated fat can lead to obesity, a primary factor in the development of IR; heme iron, advanced glycation end products, and specific amino acids (like leucine), all of which can influence how insulin is secreted [106].

### 2.10. Ethanol’s Double Edge: Systemic Insulin Resistance and Localized Urothelial Damage

The relationship between alcohol consumption and BC risk is complex and research findings can be inconsistent. Chronic heavy alcohol consumption may contribute to the development of T2DM by causing pancreatic beta-cell dysfunction, possibly by decreasing the expression of glucokinase, and/or by reducing insulin sensitivity through the inhibition of the insulin receptor [107].

Analyzing nine prospective cohort studies including 1,971,396 individuals no consistent evidence was found to definitively link alcohol consumption to an increased risk of BC [108], even though alcohol is a risk factor for obesity [109]. Other studies suggest a nuanced relationship. One meta-analysis found a 23% increase in BC risk in men [110]. The same meta-analysis by Lao et al. specifically highlighted that consumption of alcohol from liquor or spirits was associated with an increased risk of BC, with a dose–response relationship (i.e., a one-drink increment per day from liquor/spirits increased risk by 9%). Some studies have shown no association with wine consumption, while beer consumption has even been associated with a reduced risk in one study [111]. Research in East Asian populations suggests that individuals with inactive ALDH2 alleles, which affect alcohol metabolism and lead to higher acetaldehyde exposure (a known carcinogen), may have an increased risk of BC with alcohol consumption [112].

### 2.11. Exercise as a Metabolic Regulator: Breaking the Insulin Resistance–Bladder Cancer Cycle

Physical activity is associated with decreased risk of BC according to a systematic review and meta-analysis comprehending 15 studies with 5,402,369 subjects and 27,784 BC cases [113]. While the exact biological ways physical activity prevents BC are not fully understood, research suggests it works through several mechanisms. It may boost the body’s ability to remove cancer-causing substances, repair damaged DNA, and regulate cell growth, specialization, and programmed cell death. Physical activity decreases visceral fat, which in turn ameliorates chronic, low-grade inflammation leading to IR [114]. Preventing obesity and T2DM is an effective strategy for reducing the risk of BC, as both conditions are established risk factors. Beyond these direct effects, physical activity might also offer indirect protection by helping people reduce smoking [115]. Understanding the connections between metabolic health, physical activity, and cancer requires a crucial appreciation for the role of mitochondria [116].

### 2.12. Metabolic Crosstalk: How Gut and Urinary Microbiota Modulate Insulin Sensitivity and Bladder Health

Growing evidence shows the human microbiota drives cancer development through three main pathways: promoting inflammation; migrating to distant tissues; producing DNA-damaging genotoxins. This microbial risk combines with the danger posed by environmental pollutants. Enzymes like cytochrome P450 metabolize these pollutants, either activating them into potent carcinogens or deactivating them [117]. Proving a direct causative link between specific microbes or microbial patterns and BC remains a significant challenge, despite observed associations [118].

The gut-bladder axis has recently become a key area of focus in BC research. This inter-organ communication is also facilitated by neural pathways, such as vagal signaling, and by shared receptors like the farnesoid X receptor and toll-like receptor 4 [119].

The urinary microbiome engages in complex inter-system crosstalk, notably with the gut microbiome [120]. Current literature review pinpoints that the core mechanisms the regulation of SCFAs and gut hormones, both of which are critical modulators of glucose metabolism and inflammation, influencing IR [121].

A systematic review of 27 studies, which included 926 BC patients and 412 control individuals, found that the composition of urinary microbiota is altered in patients with BC. However, the studies varied significantly in their findings regarding the specific types of bacteria that were different [122]. On the other hand, population-level studies consistently demonstrate a significant reduction in the abundance of the probiotic genus *Bifidobacterium* within the gut microbiota of current smokers (high-risk subjects) compared to non-smokers, a phenomenon observed across diverse ethnic groups [123]. Research has revealed a mechanism where the metabolism of carcinogens by gut microbiota may promote chemically induced carcinogenesis in the bladder. To investigate this link, the scientists utilized a standard mouse model of BC, which is reliably induced by chronic exposure to N-butyl-N-(4-hydroxybutyl)-nitrosamine, examining the microbiota’s impact on both carcinogenesis and the compound’s toxicokinetics [124]

## 3. Discussion

First of all, it should be emphasized that BC is typically caused by a combination of risk factors consisting in the existence of a latency period (years or decades) between exposure to carcinogens and the development of BC and an inter-individual variation in the susceptibility to BC based on genetic background, lifestyle, and environmental exposures. The reason IR is frequently cited as a “universal” mechanism across various cancers (like breast, colorectal, and pancreatic) is that it creates a systemic environment that is essentially “pro-growth”.

Recent evidence characterizes IR as the unifying pathophysiological “hub” that links disparate risk factors—such as central obesity, smoking, and sedentary behavior—to bladder carcinogenesis. Many of the risks attributed to “obesity alone” are actually mediated by the underlying IR. When studies adjust for markers of IR (like HOMA-IR, fasting insulin levels), the independent effect of BMI on cancer risk often diminishes [125,126]. Therefore, IR is the central metabolic risk factor because it is the active, pathophysiological driver of the pro-growth, pro-inflammatory, and pro-angiogenic environment that BC cells need to initiate, thrive, and progress. Researchers are pushing into new areas to treat IR, whose primary causes are: chronic consumption of high-calorie diets, particularly those rich in refined carbohydrates and saturated fats, leads to excessive nutrient influx that overwhelms cellular metabolic capacity and impairs insulin signaling; elevated levels of circulating free fatty acids interfere with insulin receptor substrate (IRS) phosphorylation, disrupting the normal cascade of insulin-mediated glucose uptake in muscle and adipose tissue [127,128]; ectopic fat accumulation in non-adipose tissues such as liver, muscle, and pancreas creates lipotoxic conditions that directly impair insulin signaling pathways through the formation of toxic lipid metabolites like diacylglycerols and ceramides [129]; chronic low-grade inflammation, characterized by elevated pro-inflammatory cytokines including TNF-α, IL-6, and IL-1β, activates serine kinases that phosphorylate IRS proteins at inhibitory serine residues rather than activating tyrosine residues [130]; adipose tissue dysfunction in obesity leads to aberrant secretion of adipokines, with decreased adiponectin and increased resistin and leptin levels that collectively promote inflammatory signaling and IR [131]; activation of the inflammasome pathway and nuclear factor-kappa B (NF-κB) in metabolic tissues creates a self-perpetuating cycle of inflammation that progressively worsens insulin sensitivity [132]; mitochondrial dysfunction reduces oxidative capacity and leads to incomplete fatty acid oxidation, resulting in accumulation of lipid intermediates that activate protein kinase C isoforms which inhibit insulin signaling [133]; endoplasmic reticulum stress triggered by nutrient excess activates the unfolded protein response, which includes kinases like JNK and IKK that directly phosphorylate IRS proteins at inhibitory sites [134,135]; oxidative stress and excessive ROS production damage cellular components and activate stress-sensitive kinases that interfere with normal insulin receptor signaling cascades [136]; genetic polymorphisms in genes encoding insulin receptor, IRS proteins, glucose transporters, and enzymes involved in glucose metabolism can predispose individuals to reduced insulin sensitivity [137,138]; epigenetic modifications including DNA methylation and histone acetylation patterns altered by environmental factors can silence genes essential for proper insulin signaling or activate pro-inflammatory pathways [139]; growth hormone excess and elevated glucagon levels create counter-regulatory hormonal environments that oppose insulin’s metabolic effects and promote hepatic glucose output [140,141]; disrupted circadian rhythms and sleep deprivation alter the temporal coordination of metabolic processes, leading to desynchronization of insulin secretion and peripheral insulin sensitivity [142]; dysbiosis of the gut microbiome alters the production of short-chain fatty acids and increases intestinal permeability, allowing bacterial endotoxins like lipopolysaccharides to enter circulation and trigger systemic inflammation [143,144]; sedentary lifestyle reduces skeletal muscle glucose transporter-4 (GLUT4) expression and impairs muscle capillary density, diminishing the tissue’s capacity for insulin-mediated glucose disposal [145]; aging is associated with progressive accumulation of senescent cells that secrete inflammatory factors, coupled with declining muscle mass and increased visceral adiposity, all contributing to deteriorating insulin sensitivity [146].

High dietary glycemic load and excessive sucrose intake drive chronic hyperinsulinemia, ultimately attenuating insulin signaling and inducing systemic IR [147].

Epidemiological evidence suggests associations of high dietary glycemic index/load and high sugar consumption with BC risk [148]. An imbalance in the gut microbiome is a key factor in cancer development and progression. This disruption affects the ecosystem of gut, altering immune function and metabolism. These changes can create an environment that promotes tumor growth [149,150].

IR is closely associated with visceral adipose dysfunction and systemic inflammation4, both of which favor creating an environment conducive to tumorigenesis [151]. Additionally, epigenetic modifications which are triggered by IR and other environmental factors and chronic disease are often involved in oncogenesis, such as DNA methylation, histone modifications, and non-coding RNA [152,153]. Furthermore, the MAPK insulin pathway is the basis of many obesity-related malignancies that control cell growth and mitosis [154], whereas insulin can directly promote cell proliferation and survival via the PI3K/Akt and Ras/MAPK pathways [155].

The molecular initiation events are summarized in Table 3.

**Figure 1:**
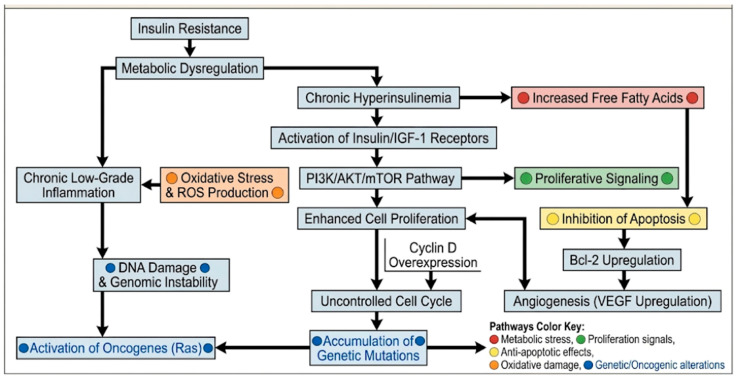
Insulin resistance and cancer: a mechanistic flowchart. VEGF, vascular endothelial growth factor; IGF, insulin growth factor. Arrows (↓, →, ←): indicate the direction of influence or progression. Downward arrows show the main cascade; side arrows represent interactions or feedback loops. The thematic categorization of the illustrated pathways is delineated by a specific color-coding system. The development of a pro-tumorigenic microenvironment is further facilitated by epigenetic reprogramming and metabolic remodeling; wherein systemic stressors induce localized phenotypic shifts that support immune evasion and stromal restructuring.

In the presence of comorbidities characterized by a chronic low-grade inflammatory state—such as obesity, rheumatoid arthritis, inflammatory bowel disease, or metabolic syndrome—the oncogenic cascade illustrated in Figure 1 would be expected to initiate earlier and progress more aggressively, as the pre-existing elevation of pro-inflammatory mediators would bypass the initial steps of insulin resistance-driven inflammation and converge directly onto the DNA damage, genomic instability, and tumor microenvironment development nodes, effectively lowering the threshold for malignant transformation.

Medications that can reduce IR with their downstream signaling events are shown in Table 4.

Obesity is a major risk factor for cancer risk primarily because it is the most common cause of IR. For effective diagnosis and treatment, clinicians must distinguish between IR linked to high adiposity and IR arising from other independent causes. This differentiation supports a precision medicine approach, allowing healthcare providers to move beyond a simple diagnosis of IR and tailor interventions based on the primary driver, whether it is systemic inflammation from excess adipose tissue or a distinct metabolic or genetic disorder. Just like with other cancers linked to obesity, weight loss is a core component of BC care through diet, exercise, weight reduction surgery, behavior therapy, and drug therapy. Noteworthy, IR is a fundamental pathophysiological mechanism underlying T2DM [177]. The systemic nature of IR suggests that regardless of the organ, the tumor is essentially hijacking a systemic state of nutrient excess and growth-factor signaling. By viewing IR as a systemic engine of malignancy, physicians move toward a model where metabolic stabilization (through diet, exercise, or insulin-sensitizers) becomes a foundational pillar of oncology, aiming to “starve” the tumor of its growth signals while restoring the host’s physiological balance. Specifically, dipeptidyl peptidase-4 inhibitor, GLP-1AR and sodium-glucose cotransporter-2 inhibitor are not significantly associated with an increased risk of BC in T2DM patients [178,179]. What is more, recent clinical data demonstrate that GLP-1RAs improved some cancer-specific outcomes and augmented response to immunotherapy. Mechanistic preclinical evidence suggests this benefit is confined to an obesity context and is mediated by the immune modulation of the tumor microenvironment [180,181]. The most common and well known side effects include nausea, vomiting, constipation, and diarrhea, which occur in up to 40% of people taking GLP-1RA drugs [182]. Adding cannabis or cannabinoids to standard antiemetic regimens may effectively reduce nausea and vomiting in patients where chemotherapy treatment has otherwise been unsuccessful [183]. Cannabidiol, interestingly, induces cell death in BC, a process further optimized by advanced intravesical delivery strategies that improve drug adhesion [184].

However, other studies on GLP-1AR have suggested a potential increase in the risk of certain cancers, including BC, though the evidence is not conclusive and requires further investigation [185]. A recent meta-analysis comprehending a total of 9 retrospective cohort studies with 1,270,179 patients revealed that metformin intake was associated with an increased recurrence-free survival (HR = 0.55), improved progression-free survival (HR = 0.70), and prolonged cancer-specific survival (HR = 0.57), indicating that metformin intake could improve the prognosis of BC patients [186]. The association between long-term GLP-1RA use and a slightly higher cancer incidence is currently viewed with caution [187].

Some research has suggested that the use of the T2DM medicine pioglitazone might be linked with an increased risk of BC. The risk seems to get higher when higher doses are used [188]. A “higher” intake of fluids and foods like fruits, vegetables, yogurt, whole grains, and dietary fiber is linked to a reduced risk of BC. This preventive effect is attributed to nutrient-rich foods, particularly fruits and vegetables, which can modulate numerous signaling pathways. For example, apples may modulate pathways related to apoptosis, proliferation, cell growth, and mitotic catastrophe. Similarly, pomegranate can modulate pathways for angiogenesis, immune response, cell proliferation, glycolysis, and the cell cycle, in addition to apoptosis [189]. Citrus fruits also contribute by modulating several pathways, including ROS production and the inhibition of cell growth and the cell cycle [190]. A current meta-analysis comprehending 12 studies showed that Mediterranean diet has protective effects on BC risk, although more research is needed to confirm the findings [191]. Combining structured exercise with dietary support is more effective for weight loss than either method alone. This approach also has the strongest positive effect on blood biomarkers linked to common cancers, such as IR and inflammatory markers [192].

### Metabolic-Immune Crosstalk and PD-L1 Modulation

The intricate interplay between the tumor microenvironment (TME), chronic low-grade inflammation, and systemic metabolic dysfunction significantly modulates the efficacy of contemporary immunotherapies in advanced BC. Chronic inflammation, often driven by insulin resistance and adipokine dysregulation, fosters an immunosuppressive TME characterized by the upregulation of programmed death-ligand 1 (PD-L1) on both malignant cells and infiltrating immune cells. This adaptive immune resistance directly hinders T-cell-mediated antitumor responses, making PD-L1 expression a critical clinical biomarker for treatment with immune checkpoint inhibitors (ICIs) such as pembrolizumab or atezolizumab. Emerging evidence suggests that GLP-1RA may offer a dual therapeutic benefit by not only stabilizing systemic metabolic parameters but also potentially attenuating the pro-inflammatory signaling pathways, such as NF-κB, that drive PD-L1 overexpression. By mitigating the inflammatory “soil” that supports immune evasion, these metabolic interventions may enhance the clinical relevance and durable response rates of ICIs in metabolically burdened patients [193].

## 4. Conclusions

IR appears to be less of a stand-alone risk and more of a common mechanism that helps connect other risk factors for BC. Conditions that promote the development of IR are often linked to BC because IR causes chronic hyperinsulinemia, which over-activates the PI3K/AKT/mTOR pathway to drive uncontrolled cell proliferation and suppress apoptosis. This metabolic state simultaneously generates oxidative stress and inflammation, leading to DNA damage and genetic mutations. The combination of sustained growth signals and accumulating mutations inactivates tumor suppressors (i.e., p53) and activates oncogenes (i.e., Ras). These processes converge to promote angiogenesis and create a tumor microenvironment contributive to cancer development. By integrating a critical discussion of cross-study findings and the overarching role of IR as a central pathophysiological driver, this manuscript provides a cohesive answer to the research question while bridging the gap between systemic metabolic health and localized urothelial carcinogenesis. Given its role as a key biological bridge, managing IR is proposed as a promising avenue to potentially reduce overall BC risk. Shifting the focus from merely describing this mechanism toward developing actionable, prescriptive IR-based risk stratification strategies could potentially improve their adoption into clinical practice and increase their real-world utility. It is important to acknowledge the inherent heterogeneity in the available literature, where findings often diverge based on study design, population demographics, and the specific metabolic markers analyzed. While the link between certain carcinogens is definitive, the precise degree to which systemic factors like IR contribute remains a subject of ongoing debate. While this review synthesizes evidence across a broad range of risk factors—from well-established exposures such as smoking and occupational chemicals to emerging domains including microplastics, air pollution, chlorinated water, sleep disruption, and microbiome alterations—it is important to acknowledge that the strength of evidence varies considerably across these areas.

IR is proposed as a convergent biological mechanism linking many of these factors to BC risk; however, this framing reflects a working hypothesis rather than an established causal pathway. Much of the evidence presented remains associative, and the mechanistic connections—though biologically plausible—are largely hypothesis-generating at this stage. Accordingly, the translational implications for prevention and clinical application should be interpreted with appropriate caution, and future research should prioritize longitudinal designs and mechanistic studies capable of moving beyond association toward a more definitive understanding of causality.

## 5. Future Directions

The association between obesity and BC is complex and actively debated in the scientific community, but the evidence linking anthropometric measures of obesity to an increased risk of developing BC is accumulating and generally supported by a causal biological mechanism. Results from a dose–response meta-analysis are associated with linear-increased risk of BC [18]. The central finding of a very recent meta-analysis is a clear association between metabolic syndrome (comprehending IR) and its individual components and an elevated risk of developing BC [194]. The TyG index, a surrogate marker for IR is a significant predictor of BC presence, outperforming other metrics, provides direct evidence that IR itself is a powerful independent risk factor [195]. To confirm previous data, prospective studies should be conducted to better understand the temporal and causal relationship.

A retrospective study conducted in 2020 on hospitalized patients found that the occurrence of Non-Alcoholic Fatty Liver Disease (NAFLD), recently renamed MASLD, was higher in patients with BC compared to those in a control group [196]. The link between NAFLD and BC is part of broader association between NAFLD and various extra-hepatic cancers, often attributed to an underlying metabolic dysregulation, namely IR, which they share [197]. There is evidence linking hepatic lipid accumulation to the development of IR, including the accumulation of triacylglycerol and lipid metabolites, such as diacylglycerol and ceramides [198]. Animal studies on Bifidobacteria have shown promising results in improving intestinal barriers, immune function, and metabolism [199]. Future research should investigate if probiotics, especially when combined with prebiotics, and a healthy lifestyle, by improving lipid profile, inflammatory and oxidative markers, SFAs production and microbiota composition, can effectively reduce IR in people [200], in the light of recent surprising findings that early metabolic changes with weight loss in humans, i.e., improving insulin sensitivity, are unlikely to be mediated by changes to the gut microbiome [201]. In this context, the Mediterranean diet raises as a highly effective approach for greater improvement of IR in obese individuals, and long-term obesity management, often exceeding the efficacy of other popular dietary patterns, including low-fat and low-carbohydrate regimens [202,203].

Both sleep physicians and urologists should further clarify the relationship between spleen disorders and BC to ensure appropriate patient management and improved outcomes.

A recent systematic review and meta-analysis, encompassing eleven multiadjusted observation studies, reveals that OSA is associated with an elevated risk of BC. While initial findings suggest OSA does not negatively impact prognosis, available mortality data remain scarce [100]. While stepwise analysis identified obesity as the main driver of IR, sleep-disordered breathing indices were also shown to be independent predictors of IR. Once again, the link between OSA and IR held true regardless of the patient’s obesity status [204]. It is now a well-established fact that people with OSA have a significantly higher likelihood of also having T2DM, and vice versa [205].

Further research is required to elucidate the specific mechanisms linking smoking to IR. Studies show that smoking cessation leads to a reduction in chronic low-grade inflammation and increased levels of adiponectin, suggesting a potential pathway for improved insulin sensitivity [206]. Alcohol is considered a carcinogen because of its metabolite, but there was no association between genetically predicted alcohol assumption and BC risk [207]. Deepening its role as risk co-factor of smoking for BC is crucial, as well as clarifying that “excessive” fluid intake may increase the risk of BC [208]. The effects of arsenic in drinking water are highly gender-specific, demonstrating a critical gap in research that must be addressed. Disparities begin with higher exposure for women through household water management duties. Susceptibility could be further influenced by biological factors, including hormonal control over metabolism in women, genetic differences in arsenic biotransformation, and the effect of nutritional status on metal absorption [209]. Compared to insufficient concentrations, high concentrations of total and estimated free 25(OH)D were found to be significantly associated with a diminished risk of BC [210]. Vitamin D supplementation significantly improved both insulin secretion and sensitivity, according to the results [211]. To confirm the positive effect of this therapeutic approach in BC patients, further investigation via well-designed clinical trials is warranted.

For non-muscle-invasive bladder tumors, downregulated adiponectin expression was an independent predictor of recurrence. Conversely, for muscle-invasive bladder tumors, upregulated leptin expression independently predicted progression [163]. Although a direct adiponectin therapy, as an antagonist of IR, is not yet available, researchers are developing therapeutic strategies using peptide or small-molecule agonists to mimic its beneficial effects. To provide a deeper clinical contextualization, this review elucidates how proposing IR as a meaningful determinant of BC risk and progression might fundamentally reshape contemporary surveillance and treatment frameworks, particularly for metabolically compromised patients. In these high-risk individuals, the systemic interplay between hyperinsulinemia and urothelial malignancy necessitates a more integrated approach to management, moving beyond traditional localized care to address the patient’s broader metabolic profile. Consequently, there is an urgent clinical need for the implementation of sensitive, non-invasive follow-up tools that can accurately monitor disease status without the morbidity of frequent invasive procedures, as highlighted by recent advancements in diagnostic methodology [212]. By adopting such specialized surveillance strategies, clinicians can better tailor interventions to the unique physiological landscape of the metabolically burdened patient, potentially improving long-term oncological outcomes.

## Figures and Tables

**Table 1:** The pathophysiological landscape: risk factors and their convergent biochemical pathways bladder cancer and mechanistic biochemical levels.

Category	Risk Factor	Description	Mechanisms
Modifiable	Smoking (tobacco use)	The leading risk factor; accounts for ~50% of cases. Carcinogens in smoke (i.e., nitrosamines, polycyclic aromatic hydrocarbons) are absorbed and excreted in urine, damaging bladder epitelium.	DNA damage and mutations in TP53 and RB1 genes [34]. Activation of MAPK/ERK pathway (promotes cell proliferation) [35]. Oxidative stress and inflammation via NF-κB pathway [36].
Modifiable	Occupational chemical exposure	Exposure to aromatic amines (i.e., benzidine, 2-naphthylamine) in industries like dye, rubber, leather, and painting. These chemicals are metabolized into bladder carcinogens.	Metabolic activation via cytochrome P450 enzymes and N-acetyltransferase polymorphisms [37]. DNA adduct formation leading to mutations in FGFR3 and HRAS genes [38]. PI3K/AKT pathway dysregulation (enhances cell survival) [39].
Modifiable	Arsenic in drinking water	Chronic exposure from contaminated water (i.e., in certain regions like parts of Asia and South America). Arsenic is a known carcinogen.	Induction of oxidative stress and DNA hypomethylation. Activation of EGFR and MAPK pathways (promotes angiogenesis and proliferation). Inhibition of DNA repair pathways (base excision repair) [40].
Modifiable	Chronic bladder irritation/Infections	Repeated urinary tract infections, bladder stones, or long-term catheter use; also linked to parasitic infections like schistosomiasis (common in Africa/Middle East).	Chronic inflammation via NF-κB and COX-2 pathways (leads to squamous cell carcinoma) [41]. Nitrosamine formation from nitrates in urine, causing DNA alkylation [42]. Upregulation of STAT3 pathway (enhances immune evasion and tumor growth) [43].
Modifiable	Low fluid intake/Dehydration	Insufficient water consumption leads to concentrated urine, prolonging exposure to carcinogens in the bladder.	Increased concentration of urinary carcinogens, amplifying DNA damage. Indirect activation of oxidative stress pathways (ROS-mediated damage) [44]. Potential link to altered metabolic pathways like urea cycle dysregulation [45].
Modifiable	Certain medications/Treatments	Prior use of cyclophosphamide (chemotherapy) or pelvic radiation therapy for other cancers.	Alkylating agents cause DNA cross-links and mutations in TP53. Radiation induces double-strand breaks, activating ATM/ATR DNA damage response pathways. PI3K/AKT/mTOR pathway overactivation (promotes cell survival post-damage) [46].
Non-Modifiable	Age	Risk increases significantly after age 55; ~90% of cases occur in people over 55.	Accumulation of somatic mutations over time (i.e., in FGFR3 and TERT genes). Age-related decline in DNA repair pathways (nucleotide excision repair). Telomere shortening and senescence bypass via p16/RB pathway [47].
Non-Modifiable	Gender (Male)	Men are 3–4 times more likely to develop BC, possibly due to higher smoking rates and occupational exposures historically.	Hormonal influences (androgen receptor signaling may promote tumor growth) [48]. Genetic factors like X-chromosome inactivation in females providing protective effects [49]. Interaction with smoking-related pathways (enhanced CYP1A1 metabolism in males) [50].
Non-Modifiable	Family history/Genetics	Inherited predisposition (i.e., Lynch syndrome or polymorphisms in genes like GSTM1, NAT2). Family history increases risk by 1.5–2 times.	Germline mutations in mismatch repair genes (MSH2 in Lynch syndrome), leading to microsatellite instability [51]. Polymorphisms in detoxification pathways (GST and NAT enzymes affecting carcinogen metabolism) [52]. Hereditary activation of Wnt/β-catenin or RAS pathways [53].
Non-Modifiable	Race/Ethnicity	Higher incidence in White populations compared to Black or Asian; possibly due to genetic and environmental factors.	Genetic variations in drug-metabolizing enzymes (CYP2D6 polymorphisms) [54]. Epigenetic changes influencing pathways like histone modification. Interaction with environmental exposures amplifying TP53 mutations [55].

BC, bladder cancer. ROS, reactive oxygen species. Numerous risk factors converge on a set of shared molecular pathways that drive the development and progression of BC. A critical early step involves the disruption of the DNA damage response, where alterations in the TP53 and RB1 genes lead to a profound loss of cell cycle control. This is frequently accompanied by aberrant oncogenic signaling; while FGFR3 mutations are characteristic of non-muscle-invasive bladder cancer, the PI3K/AKT pathway often predominates in more aggressive, muscle-invasive types. Furthermore, chronic inflammation—driven primarily by the NF-κB pathway—creates a persistent state of irritation that facilitates malignant transformation. On a systemic level, metabolic activation via liver enzymes, such as the CYP450 family, is essential for converting inert environmental exposures into active carcinogens that specifically target the bladder urothelium. This pro-tumorigenic environment is further sustained by a complex network of signaling mediators, including hypoxia-inducible factor 1 (HIF-1), insulin-like growth factor receptor 1 (IGF-1R), and Wnt signaling, alongside various dysregulated cell cycle proteins that fuel uncontrolled cellular proliferation. While many of these metabolic and signaling links remain associated, the evidence is most robust for smoking and chemical exposures, which are firmly established as the strongest drivers of the disease by large-scale cohort studies [56].

**Table 2:** Summary of lifestyle and environmental risk factors, associated pathologies, and biochemical pathways.

Risk Factor	Associated Diseases/Conditions	Primary Biochemical Pathway(s) Affected	Mechanism/Pathological Result
High Glucose/Hyperglycemia	Type 2 Diabetes, Cardiovascular disease, Neuropathy	Polyol Pathway; Hexosamine Pathway; AGE Formation	Excess glucose is shunted into the polyol pathway, depleting NADPH and increasing oxidative stress. It also forms AGEs that cross-link proteins and damage blood vessels.
Obesity/ Visceral Adiposity	Metabolic Syndrome, Type 2 Diabetes, NAFLD	PI3K/Akt Insulin Signaling Pathway; JAK/STAT Pathway	Hypertrophied fat cells secrete pro-inflammatory cytokines (TNF-α, IL-6) that cause serine phosphorylation of IRS-1, inhibiting the PI3K/Akt pathway and inducing systemic insulin resistance.
Chronic Smoking	COPD, Atherosclerosis	Aryl Hydrocarbon Receptor (AhR); NF-κB Pathway; CYP450 Enzymes	Polycyclic aromatic hydrocarbons activate AhR, upregulating CYP1A1 (leading to DNA-damaging reactive metabolites). Simultaneously, toxins activate NF-κB, driving chronic inflammation.
Excessive Alcohol Intake	Alcoholic Liver Disease, Cirrhosis, Chronic Pancreatitis	Ethanol Oxidation (ADH/ALDH); CYP2E1 Pathway	Metabolism of ethanol increases the NADH/NAD+ ratio, halting fatty acid oxidation (causing fatty liver). CYP2E1 induction generates massive Reactive Oxygen Species (ROS), causing hepatic oxidative stress.
Sedentary Lifestyle	Obesity, Cardiovascular disease, Sarcopenia	AMPK Signaling; PGC-1α (Mitochondrial Biogenesis)	Lack of cellular energy stress keeps AMPK inactive, reducing GLUT4 translocation (impaired glucose uptake) and fatty acid oxidation. Downregulation of PGC-1α leads to decreased mitochondrial density and function.
High Saturated Fat Intake	Atherosclerosis, Hyperlipidemia, Alzheimer’s Disease	Cholesterol Biosynthesis (HMG-CoA Reductase); TLR4 Signaling	Upregulates endogenous cholesterol synthesis. Saturated fatty acids bind to TLR4 on macrophages, activating the NF-κB pathway and promoting vascular inflammation and plaque formation.
Chronic Psychological Stress	Hypertension, Depression, Immune suppression	(HPA) Axis; Catecholamine Synthesis	Sustained cortisol secretion alters tryptophan metabolism (shifting it toward the kynurenine pathway, neurotoxic) and chronically activates the sympathetic nervous system, increasing blood pressure and suppressing lymphocyte proliferation.
Aging (Cellular Senescence)	Neurodegeneration, Frailty	mTOR Signaling; SIRT1 Pathway; p53/p21	Accumulated DNA damage activates p53/p21, leading to cell cycle arrest. Decreased NAD+ availability blunts SIRT1 activity (reducing DNA repair and mitochondrial efficiency), while overactive mTOR inhibits cellular autophagy (clearance of cellular debris).
Environmental Toxins (e.g., Heavy Metals, BPA)	Neurotoxicity, Endocrine disruption	GSH Metabolism; ER Pathway	Heavy metals bind to sulfhydryl groups, severely depleting glutathione and disabling antioxidant defense. Endocrine disruptors (like BPA) act as xenoestrogens, aberrantly activating the ER pathway and altering gene transcription.
Vitamin D Deficiency	Osteoporosis, Autoimmune diseases, Depression	Calcium/Phosphorus Homeostasis; VDR Signaling	Lack of active calcitriol fails to activate the Vitamin D Receptor, a transcription factor responsible for expressing hundreds of genes, including those regulating immune tolerance (Th1/Th2 balance) and osteoclast/osteoblast regulation.

Adding comorbidities transforms the static molecular diagram into a dynamic clinical roadmap. It acknowledges that BC does not occur in a vacuum and that effective prevention, diagnosis, and treatment must account for the patient’s entire biological and medical context. Mechanistic Overlap: several risk factors (e.g., High Glucose, Saturated Fat Intake, and Smoking) converge on the activation of inflammatory pathways, particularly NF-κB, highlighting the commonality of chronic inflammation as a systemic pathological driver. Metabolic Signaling: the pathways listed (such as AMPK, mTOR, and Insulin Signaling) represent key metabolic regulators that, when dysregulated by these risk factors, contribute to age-related diseases and systemic frailty. Abbreviations: AGEs, Advanced Glycation End-products; AhR, Aryl Hydrocarbon Receptor; AMPK, AMP-activated protein kinase; BPA, Bisphenol A; COPD, Chronic Obstructive Pulmonary Disease; CYP450, Cytochrome P450 enzymes; GLUT4, Glucose Transporter Type; GSH, Glutathione; HMG-CoA, 3-hydroxy-3-methylglutaryl-coenzyme; AHPA, Hypothalamic–Pituitary–Adrenal (axis); IRS-1, Insulin Receptor Substrate 1; JAK/STAT, Janus Kinase / Signal Transducer and Activator of Transcription; NAFLD, Non-Alcoholic Fatty Liver Disease; NAD+/NADH, Nicotinamide adenine dinucleotide (oxidized/reduced forms); NF-κB, Nuclear Factor kappa-light-chain-enhancer of activated B cells; PGC-1α, Peroxisome proliferator-activated receptor gamma coactivator 1-alpha; PI3K/Akt, Phosphoinositide 3-kinase / Protein Kinase; BROS, Reactive Oxygen Species; SIRT1, Sirtuin 1; TLR4, Toll-Like Receptor 4; TNF-α,Tumor Necrosis Factor-alpha; VDR, Vitamin D Receptor; ER, Estrogen Receptor.

**Table 3:** Mechanisms of insulin resistance.

Molecular Mechanism	Cellular Consequence	Key Molecular Players	References
Insulin/IGF-1 signaling	Hyperinsulinemia activates insulin/IGF-1 signaling, promoting cell proliferation and survival in BC cells.	Insulin, IGF-1, IGF1R, IR	[156,157]
PI3K/AKT/mTOR pathway	IR activates PI3K/AKT/mTOR pathway, leading to increased cell growth, proliferation, and survival in BC cells.	PI3K, AKT, mTOR, PTEN	[158,159]
Inflammation and oxidative stress	IR induces chronic inflammation and oxidative stress, which can lead to DNA damage and BC initiation.	TNF-α, IL-6, NF-κB, ROS	[160,161]
Adipokine imbalance	IR alters adipokine secretion, including decreased adiponectin and increased leptin, which can promote BC cell growth and survival.	Adiponectin, leptin, adiponectin receptor	[162,163]
Epigenetic modifications	IR can lead to epigenetic changes, such as DNA methylation and histone modification, which can silence tumor suppressor genes and activate oncogenes in BC.	DNMT1, HDAC1, HAT1	[164,165]
Circadian rhythm disruption	IR can disrupt circadian rhythms, leading to altered expression of clock genes and increased risk of BC.	PER2, PER3, CRY1, CRY2	[166,167]
MicroRNA dysregulation	Insulin resistance can alter microRNA expression, including miR-21, miR-143, and miR-145, which can contribute to BC development and progression.	miR-21, miR-143, miR-145, Dicer	[168,169]
Stem cell regulation	IR can affect stem cell self-renewal and differentiation, leading to increased cancer stem cell populations and BC initiation.	OCT4, SOX2, NANOG, BMI1	[170,171]

BC, bladder cancer; IR, insulin resistance.

**Table 4:** Drugs favorably impacting insulin resistance.

Drug Class	Examples	Mechanism of Action	References
Biguanides	Metformin	Enhances glucose uptake in peripheral tissues by increasing GLUT4 expression and promoting its movement to the cell surface.	[172]
GLP-1RA	Albiglutide, Dulaglutide, Liraglutide, Semaglutide	Reduces inflammation and oxidative stress, regulates lipid metabolism, and promotes glucose transporter protein expression in insulin-dependent tissues.	[173]
SGLT2 inhibitors	Canagliflozin, Dapagliflozin, Empagliflozin, Tofogliflozin	Blocks renal glucose reabsorption, leading to increased glucose excretion. Also enhances insulin sensitivity by lowering body weight and reducing glucose toxicity.	[174]
Sulfonylureas	Glimepiride, Glipizide	Stimulates insulin receptor activity, thereby boosting glucose transporter protein numbers and improving insulin sensitivity.	[175]
Thiazolidinediones	Pioglitazone, Rosiglitazone	Improves insulin-stimulated glucose uptake, reduces pro-inflammatory cytokine production, and stimulates adiponectin release.	[176]

SGLT 2, sodium-glucose transport protein 2; GLP-1RA, glucagon-like peptide-1 receptor agonist; GLUT4, glucose transporter protein type-4.

## Data Availability

The original contributions presented in this study are included in the article. Further inquiries can be directed to the corresponding author.

## Abbreviations

BC, bladder cancer; IR, insulin resistance; GLOBOCAN, global cancer observation; BMI, body mass index; HR, hazard ratio; NMIBC, non-muscle invasive BC; IARC, International Agency for Research on Cancer; RR, relative risk; TP53, tumor protein 53; RB1, retinoblastoma protein; MAPK/ERK, mitogen-activated protein kinase/extracellular signal-regulated kinase; NF-κB, nuclear factor kappa-light-chain-enhancer of activated B cells; FGFR3, fibroblast growth factor receptor 3; HRAS, Harvey rat sarcoma virus; PI3K/AKT, phosphoinositide 3-kinase/protein kinase B; EGFR, epidermal growth factor receptor; MAPK, mitogen-activated protein kinase; COX, cyclooxygenate; STAT3, signal transducer and activator of transcription 3; ROS, reactive oxygen species; mTOR, mammalian target of rapamycin; ATM/ATR, ataxia-telangiectasia mutated/ataxia telangiectasia and Rad3-related protein; FGFR3, fibroblast growth factor receptor 3; TERT, telomerase reverse transcriptase; CYP1A1, Cytochrome P450; GST, glutathione S-transferase; NAT, N-acetyltransferase; IGF-1R, Insulin-like Growth Factor Receptor 1; MP, microplastic; BPA, bisphenol A; T2DM, type 2 diabetes mellitus; SOCS-3; suppressor of cytokine signaling 3; THM, trihalomethanes; GH, growth hormone; SDB, sleep-disordered breathing; OSA, obstructive sleep apnea; ALDH2, aldehyde dehydrogenase; SCFA; short-chain fatty acids; INSRs, insulin receptors; TNF-α, tumor necrosis factor- alpha; IL-6, interleukin-6; NF-κB, nuclear factor kappa-light-chain-enhancer of activated B cells; DNMT1, DNA-methyltransferase 1; HDAC1, histone deacetylase 1; HAT1, histone acetyltransferase 1; PER2/PER3/CRY1/CRY2, genes coding for core components of the mammalian circadian clock; OCT4/SOX2/NANOG/BMI1, embryonic stem cell transcription factors; TyG, triglycerides-glucose index; GLP-1RA, glucagon-like peptide-1 receptor agonist; TME, tumor microenvironment.
